# A conserved epitope in VAR2CSA is targeted by a cross-reactive antibody originating from *Plasmodium vivax* Duffy binding protein

**DOI:** 10.3389/fcimb.2023.1202276

**Published:** 2023-06-16

**Authors:** Uwa Iyamu, Daniel Ferrer Vinals, Bernard Tornyigah, Eliana Arango, Rakesh Bhat, Trixie Rae Adra, Simranjit Grewal, Kimberly Martin, Amanda Maestre, Michael Overduin, Bart Hazes, Stephanie K. Yanow

**Affiliations:** ^1^School of Public Health, University of Alberta, Edmonton, AB, Canada; ^2^Grupo Salud y Comunidad, Facultad de Medicina, Universidad de Antioquia, Medellín, Colombia; ^3^Grupo de Enfermedades Infecciosas y Crónicas (GEINCRO), Fundación Universitaria San Martín, Sabaneta, Colombia; ^4^Department of Biochemistry, University of Alberta, Edmonton, AB, Canada; ^5^Department of Medical Microbiology and Immunology, University of Alberta, Edmonton, AB, Canada

**Keywords:** malaria, VAR2CSA, cross-reactivity, PvDBP, peptide, vaccine design, pregnancy

## Abstract

During *Plasmodium falciparum* infection in pregnancy, VAR2CSA is expressed on the surface of infected erythrocytes (IEs) and mediates their sequestration in the placenta. As a result, antibodies to VAR2CSA are largely restricted to women who were infected during pregnancy. However, we discovered that VAR2CSA antibodies can also be elicited by *P. vivax* Duffy binding protein (PvDBP). We proposed that infection with *P. vivax* in non-pregnant individuals can generate antibodies that cross-react with VAR2CSA. To better understand the specificity of these antibodies, we took advantage of a mouse monoclonal antibody (3D10) raised against PvDBP that cross-reacts with VAR2CSA and identified the epitopes targeted by this antibody. We screened two peptide arrays that span the ectodomain of VAR2CSA from the FCR3 and NF54 alleles. Based on the top epitope recognized by 3D10, we designed a 34-amino acid synthetic peptide, which we call CRP1, that maps to a highly conserved region in DBL3X. Specific lysine residues are critical for 3D10 recognition, and these same amino acids are within a previously defined chondroitin sulfate A (CSA) binding site in DBL3X. We showed by isothermal titration calorimetry that the CRP1 peptide can bind directly to CSA, and antibodies to CRP1 raised in rats significantly blocked the binding of IEs to CSA *in vitro*. In our Colombian cohorts of pregnant and non-pregnant individuals, at least 45% were seroreactive to CRP1. Antibody reactivities to CRP1 and the 3D10 natural epitope in PvDBP region II, subdomain 1 (SD1), were strongly correlated in both cohorts. These findings suggest that antibodies arising from PvDBP may cross-react with VAR2CSA through the epitope in CRP1 and that CRP1 could be a potential vaccine candidate to target a distinct CSA binding site in VAR2CSA.

## Introduction

Infection with *Plasmodium falciparum* during pregnancy poses a severe health risk to the mother and infant. In 2021, over 13 million pregnant women in the WHO African region were infected, with more than 900,000 neonates at risk of low birth weight due to malaria (World Health Organization, 2022). During infection in pregnancy, infected erythrocytes (IEs) sequester in the intervillous spaces of the placenta (placental malaria), which disrupts the transfer of oxygen and nutrients from the maternal blood to the fetus, causing low birth weight, stillbirth, preterm birth, intrauterine growth restriction and maternal anemia (Bauserman et al., 2019).

The pathogenesis of *P. falciparum* placental malaria involves the adherence of IEs to chondroitin sulfate A (CSA) on the placenta syncytiotrophoblast (Fried and Duffy, 1996; Muthusamy et al., 2004; Ayres Pereira et al., 2016). The infected cells bind to CSA *via* the parasite ligand VAR2CSA (Salanti et al., 2004), a variant within the family of proteins known as *P. falciparum* erythrocyte membrane protein 1 (PfEMP1) (Hviid and Jensen, 2015). These proteins are encoded by about 60 different *var* genes with the *var2csa* variant expressed uniquely in placental parasites (Gardner et al., 2002; Salanti et al., 2003; Duffy et al., 2005; Tuikue Ndam et al., 2005). VAR2CSA is a large membrane-spanning protein with an extracellular region that consists of Duffy binding-like (DBL) domains. DBL domains are adhesive modules present in all PfEMP1s and in invasion ligands like *P. knowlesi* Duffy binding protein (PkDBP), *P. vivax* DBP, Erythrocyte binding antigen 175 (EBA-175), EBA-140, EBA-181 (Howell et al., 2006). The architecture of the PfEMP1 DBL domains likely facilitates targeted interactions with specific host receptors, including CD36, ICAM-1, and CSA in the placenta (Baruch et al., 1996; Hviid and Jensen, 2015). VAR2CSA typically contains six DBL domains (DBL1X, DBL2X, DBL3X, DBL4ε, DBL5ε, and DBL6ε) (Gardner et al., 2002; Hviid and Jensen, 2015), with a variant reported that contains seven (Doritchamou et al., 2019). These DBL domains are interspersed by cysteine-rich interdomain regions and the overall VAR2CSA structure maintains an interwoven architecture, with the DBL domains adopting alpha-helical conformations surrounded by loops (Khunrae and Higgins, 2010; Bewley et al., 2020). The negatively charged CSA binds to a positively charged pocket in VAR2CSA formed by the N-terminal sequence (NTS), DBL1X, DBL2X, and DBL4ε domains (Ma et al., 2021; Wang K. et al., 2021; Wang W. et al., 2021). Although individual recombinant DBL domains and VAR2CSA fragments can bind to CSA *in vitro* (Dahlbäck et al., 2006; Higgins, 2008; Singh et al., 2008; Khunrae et al., 2009; Singh et al., 2010), the full ectodomain of the protein binds to CSA with higher affinity (Srivastava et al., 2010; Dahlbäck et al., 2011; Srivastava et al., 2011; Clausen et al., 2012).

VAR2CSA is expressed by parasites infecting pregnant women and antibodies to VAR2CSA are associated with improved birth outcomes (Fried and Duffy, 2017; McLean et al., 2021). Based on *in vitro* assays, these antibodies can block adherence of IEs to CSA and opsonize infected cells for destruction by phagocytes (Lambert et al., 2014; Fried and Duffy, 2017). Due to the critical role of VAR2CSA, constructs of this protein are targeted as vaccine candidates against placental malaria (Mordmüller et al., 2019; Sirima et al., 2020). The two leading vaccines, PAMVAC and PRIMVAC, comprise the domains required for binding to CSA (ID1-ID2a and DBL1X-DBL2X, respectively) (Nielsen et al., 2015; Chêne et al., 2018). In Phase I trials, both vaccines elicited strong antibodies that recognized IEs expressing the same allele of VAR2CSA as the vaccine but exhibited limited efficacy against heterologous parasites (Mordmüller et al., 2019; Sirima et al., 2020). It is likely that the vaccines elicit antibodies to immunodominant epitopes in VAR2CSA that are highly polymorphic, a common shortcoming of most malaria vaccine candidates (Stanisic et al., 2013; Gamain et al., 2021). An alternative strategy to circumvent this challenge is to focus on conserved epitopes that elicit broadly neutralizing antibodies, an approach adopted against pathogens like *Streptococcus* (Pandey et al., 2018), Ebola (Mitchell et al., 2017) and influenza (Bajic et al., 2019).

While the acquisition of antibodies to VAR2CSA is primarily parity-dependent, VAR2CSA antibodies were reported in men and children (Gnidehou and Yanow, 2021), suggesting there is an alternative mechanism for acquiring these antibodies outside of pregnancy. One plausible explanation is that VAR2CSA expression is dysregulated in certain parasites, no longer restricting expression to pregnancy. Anti-VAR2CSA antibodies could also arise from exposure to other *Plasmodium* proteins that share epitopes with VAR2CSA. In support of the latter hypothesis, we reported a high frequency of Colombian men and children with antibodies to VAR2CSA and demonstrated that exposure to the DBL-containing protein, PvDBP, can elicit antibodies that cross-react with VAR2CSA (Gnidehou et al., 2014; Gnidehou et al., 2019). Specifically, human antibodies to region II of PvDBP (DBP-II) and a mouse monoclonal antibody, MAb, (3D10) raised against DBP-II recognized VAR2CSA (Gnidehou et al., 2019; Mitran et al., 2019). Further, these cross-reactive antibodies exhibited moderate inhibitory activity in blocking binding of IEs to CSA *in vitro*, suggesting the targets of these antibodies may reveal new epitopes to be exploited in vaccine design. In this study, we screened VAR2CSA peptide arrays to map the epitopes recognized by the 3D10 MAb. We identified an epitope targeted by 3D10 that is highly conserved across diverse *var2csa* alleles. This epitope is recognized by >50% of VAR2CSA-positive sera from Colombian men and children and strongly correlates with reactivity to the related epitope in PvDBP. Of significant interest, the epitope lies within a CSA binding site in DBL3X, suggesting a mechanism to explain the inhibitory activity of PvDBP antibodies against VAR2CSA-expressing IEs.

## Materials and methods

### Study population and sample collection

Participants were recruited from two Colombian municipalities: Puerto Libertador and Tierralta. Both municipalities are within the endemic region Urabá-Bajo Cauca-Sinú-San Jorge, which reports the second highest number of malaria cases in Colombia (Padilla-Rodríguez et al., 2021). The Department of Córdoba contributes around 13% of malaria cases in Colombia and Tierralta is the municipality with the highest number of malaria-infected people (Helías Rodríguez, 2021). Around 70% of malaria cases in Córdoba are caused by *P. vivax*. The entomological inoculation rate ranged from 3.5 to 4.8 infective bites per person per year; the annual parasite index has been >25 per 1,000 exposed residents since 1950; and malaria transmission is not seasonal (Carmona-Fonseca, 2017).

In Puerto Libertador, asymptomatic and symptomatic non-pregnant participants (males over 5 years and females from 5-12 years old) were recruited between 2013 and 2016 (Gnidehou et al., 2019). The symptomatic participants were recruited at a local clinic while the serum samples from asymptomatic participants were collected during a community-based cross-sectional survey. Also, samples were collected from pregnant women recruited at an antenatal care clinic during a longitudinal study (Gavina et al., 2018). In Tierralta, 93 volunteers (males over 5 years and females from 5-12 years old) were recruited in February to March 2022. All volunteers were recruited from the community and were mostly asymptomatic, with 10 volunteers having malaria symptoms. During that period, 3,828 cases of malaria were reported by the surveillance system of Colombia; of those cases, 62% were with *P. vivax*, and 15% were reported in Córdoba. Twelve sera were collected as negative controls from residents living in Medellín, a municipality with no malaria cases. As a positive control for serological assays, we used a pool of sera from multigravid women residing in Jinja, Uganda. For all participants, blood samples (4-5 ml) were collected by venipuncture as described previously (Gnidehou et al., 2019).

### Generation and analysis of peptide arrays

Arrays of synthetic peptides were designed to span the extracellular domain of VAR2CSA from the strains FCR3 (GenBank: AAQ73926.1, accession: AY372123.1, residues 1-2659) and NF54 (GenBank: EWC87419.1, accession: KE123842.1, residues 1-2652). Peptides were 20 amino acids in length with an overlap of 19 and 18 amino acids for the FCR3 or NF54 arrays, respectively. Arrays were synthesized by PepperPrint™ (Germany) and screened with the 3D10 MAb at 10 μg/ml. Each peptide was tested in duplicate.

Raw data from the peptide microarrays were analyzed with a Python script “plotepiscan.md”, built on top of the Python package Biotite, version 0.35.0 (Kunzmann and Hamacher, 2018). Signal intensities for each peptide were based on the average between median foreground intensities of each spot. We tolerated a maximum replicate-to-replicate deviation of 40%; otherwise, the intensity value computed was selected from the replicate with the highest score value. Intensity data were normalized using a cubic transformation implemented in the plotepiscan script. These data were mapped onto an alignment between the FCR3 and NF54 alleles. We highlighted antibody recognition on each peptide array using a color code from red to white for high to low intensity, respectively. Each color corresponds to the score for the 20 amino acid peptide that ends at that residue.

For epitope selection we considered the most reactive regions according to the intensity color code and that showed consistency in 3D10 recognition against the same region in both the FCR3 and NF54 alleles. To account for the 19-mer offset of the highlighted residues, we included the immediate 19 “upstream positions” (from C to N-terminus) preceding the residue with the highest score. All data files are freely available in the GitHub repository (https://github.com/Yanow-lab/Iyamu-et-al).

### Sequence alignment and logo plot

Sequence data from field isolates were available from the *P. falciparum* Community Project accessible from the Pf3k website (https://www.malariagen.net/projects/pf3k). We included VAR2CSA sequences from field isolates from the following countries: Ghana, Cambodia, Guinea, Nigeria, Thailand, Malaysia, Kenya, Bangladesh, Gambia, Congo, Mali, Senegal, Vietnam, Myanmar, and Laos. (n=722). The raw sequence data are available in the SRA studies ERP000190 and ERP000199. We also included the sequences from the lab-adapted strains FCR3 and NF54. A multiple sequence alignment was prepared with Clustal Omega (Sievers and Higgins, 2021) using the EMBL-EBI online server and default parameters (Madeira et al., 2022). Aligned sequences were visualized with Jalview (version 2.11.2.1) and a logo plot was generated using the WebLogo server with default parameters (Crooks et al., 2004).

### Synthetic peptides and recombinant proteins

The sequence information for synthetic peptides generated by Synpeptide Co., China, is provided in **Table 1**. SD1_CLIPS_ contains the SD1 region of PvDBP (Sal1): C(T2-013)NYKRKRRERDWDCNTKKDVCIPDRRYQLC(T2-013)K(Aoa) (synthesized by Biosynth), and was constrained using CLIPS (Chemically Linked Peptides on Scaffolds) technology (Timmerman et al., 2007; Mitran et al., 2020). The recombinant VAR2CSA (FCR3) and ID1-ID2a (FCR3) were kindly shared by Dr. Ali Salanti, University of Copenhagen.

**Table 1:** Peptide information.

Peptide name	Sequence
CRP1	KKYISEKKQEWDKQKTKYENKYVGKSASDLLKEN (residues 1509-1542, FCR3)
CRP-K1510A	KAYISEKKQEWDKQKTKYENKYVGKSASDLLKEN
CRP1-AAA	KKYISEKKQEWDAQKTAYENAYVGKSASDLLKEN
Scrambled CRP1 peptide	KQIWLKSKSKNKTEEKGKDYYQKEKYKANLEVSD
P10^#^	RKSNKESEGGKDYSMIMEPTVIDYLNKRCNGEINGN
P5^*^	KRWDQIYKRYSKHIEDAKR

#Negative control for competition ELISA.

*Negative control for the rat immunization experiments.

### ELISA

#### Indirect ELISA

Immunoglobulin G (IgG) reactivity was measured using the enzyme-linked immunosorbent assay (ELISA). Optimized concentrations of reagents are provided in **Table 2**. Briefly, 96-well Maxisorb plates (catalogue no. 439454; Thermo Fisher Scientific) were coated with synthetic peptides and recombinant full-length VAR2CSA diluted in 1 x PBS and incubated overnight at 4°C. Coated plates were blocked with 4% bovine serum albumin (BSA, catalogue no. A7906; Sigma-Aldrich) for 1 h at 37°C, then washed once with 1 x PBST (0.1% Tween 20). Plates were incubated with primary antibody for 1 h at room temperature (RT) and washed four times with 1 x PBST. The washed plates were incubated at RT for 1 h with 100 µl of HRP-conjugated secondary antibody (goat anti-human HRP, catalogue no. ab98624, Abcam; goat anti-mouse HRP, catalogue no. 170-6516, Bio-Rad, Mississauga, Canada; goat anti-rat HRP catalogue no. 62-9520). After four washes, 100 µl of 3,3′,5,5′-Tetramethylbenzidine (TMB, catalogue no. T0440; Sigma-Aldrich) were added and the reaction was stopped after 30 mins with an equal volume of H_2_SO_4_ (0.5 N). Human sera were screened once, in duplicate. The optical density (OD) values were converted to arbitrary units relative to the positive control sera included on each plate using the formula: AU = OD_test_ -OD_background_/OD_positive control pool_ – OD_background_)*100. The positive control pool refers to the OD of a pool of Colombian or Ugandan samples that were seroreactive against the peptides or VAR2CSA, respectively. The background is the OD with secondary antibody alone. The cut-off for seropositivity was based on the mean AU of sera from 12 individual Colombians not exposed to malaria plus 2 standard deviations; data to generate the cut-off are from two independent experiments. Only sera that were above the cut-off for VAR2CSA seropositivity were screened against CRP1 and SD1_CLIPS_.

**Table 2:** ELISA conditions.

Antigen	Antigen concentration (µg/ml)	Primary antibody	Primary antibody dilution	Secondary antibody dilution
**CRP1**	5	Human sera	1/400	1/40,000
	1 & 5	3D10	Titration	1/3,000
	5			
		Rat sera	1/100	1/3,000
**SD1_CLIPS_**	1	Human sera	1/200	1/30,000
		3D10	Titration	1/3,000
		3D10	1.72 (µg/ml)	1/3,000
**VAR2CSA**	0.5	Human sera	1/1000	1/15,000
	1	3D10	8.6 (µg/ml)	1/3,000

#### Competition ELISA

Similar steps were followed as the indirect ELISA, except plates were blocked for at least 1.5 h, and the primary antibody was pre-incubated with 10 µg/ml of competing antigen for 1 h at RT, then added to the coated plates.

### Isothermal titration calorimetry (ITC)

Bovine tracheal CSA was purchased from Sigma-Aldrich (Cat no C9819). The energetics of the binding of peptides to CSA was performed at 25°C, on a MicroCal PEAQ-ITC calorimeter (Malvern Panalytical, Malvern, USA). For all experiments, samples were prepared in PBS containing 0.5% acetonitrile, pH 7.4. The reference cell was loaded with autoclaved milli-Q ultra-pure water; the sample cell was loaded with peptide solution (50 µM) and the syringe was filled with 1 mM CSA solution (based on a molecular weight of 50 kDa). In each titration, 25 injections of 1.5 μl of the CSA solution were made into the sample cell. A spacing of 150 s between each injection was applied to enable the system to reach equilibrium. The ITC data were analyzed with MicroCal-PEAQ-ITC Analysis Software v1.41. Binding parameters such as the Δ*H* (enthalpy change) and the Δ*S* (entropy change) were determined by fitting the experimental binding isotherms with the ‘one set of sites’ fitting model and GraphPad Prism software (version 9) was used to plot the results.

### Rat immunization experiments

CRP1 was conjugated to BSA (as carrier protein) at the N-terminus through an azide-alkyne cycloaddition and outsourced commercially to ProSci (California, USA) for rat immunizations. As controls, an unrelated peptide conjugate (P5-BSA), the PAMVAC vaccine candidate (ID1-ID2a region from VAR2CSA) and the carrier protein conjugated to the linker (azido-ethanol (AE-BSA)) were included. Briefly, two female Sprague Dawley rats (~11 weeks old) were immunized intraperitoneally on day 0 with 75 µg of each antigen formulated in PBS and Complete Freund’s Adjuvant (CFA) followed by 3 booster immunizations with 50 µg of antigen in Incomplete Freund’s Adjuvant at 2-week intervals. Sera were collected 14 days after the final boost.

### *P. falciparum* culture and purification of IEs

*P. falciparum* CS2 parasites were cultured *in vitro* as described (Trager and Jensen, 1976). Parasites were selected for adhering to CSA every 3-4 weeks to enrich for parasites expressing VAR2CSA. Expression was confirmed by flow cytometry with an anti-VAR2CSA rabbit antibody, kindly shared by Dr. Ali Salanti. IEs containing mature parasites were purified using the VarioMACS separator according to the manufacturer’s instructions (LD columns, catalogue no. 130-042-901; Miltenyi Biotec).

### Inhibition of binding assay (IBA)

The ability of anti-CRP1 IgG to block parasite binding to CSA *in vitro* was assessed by a static binding assay (Gnidehou et al., 2014). Briefly, 20 µl of CSA was spotted onto a Petri dish and incubated overnight. Spots were blocked with 3% BSA in RPMI medium for 1 h at 37°C. Erythrocytes infected with mature trophozoites (CS2 strain) were preincubated with 200 µg/ml IgG from immunized rats for 30 min at RT. Preincubation with soluble CSA (250 µg/ml) was used as a positive control for inhibition. The mixtures were added to the CSA spotted plate for 15 mins at RT. After washing, spots were fixed with glutaraldehyde and stained with Giemsa before imaging using a EVOS FL Auto microscope (Invitrogen) and the number of parasites bound per spot was analyzed using ImageJ. Percent inhibition of binding was calculated relative to the counts from the AE-BSA control.

### Statistical analyses

Data were plotted using Prism software. The normal distribution of data was first examined using the D’Agostino & Pearson test. The correlation of CRP1 and SD1_CLIPS_ serological data was determined using the Spearman correlation. For the analysis of the IBA data, percent parasite inhibition within each group was compared across all groups using the one-way ANOVA test in conjunction with Tukey’s multiple comparisons *post-hoc* tests. Statistical significance was set to *P*<0.05.

## Results

### The PvDBP-derived MAb 3D10 recognizes an epitope in DBL3X

**Figure 1:**
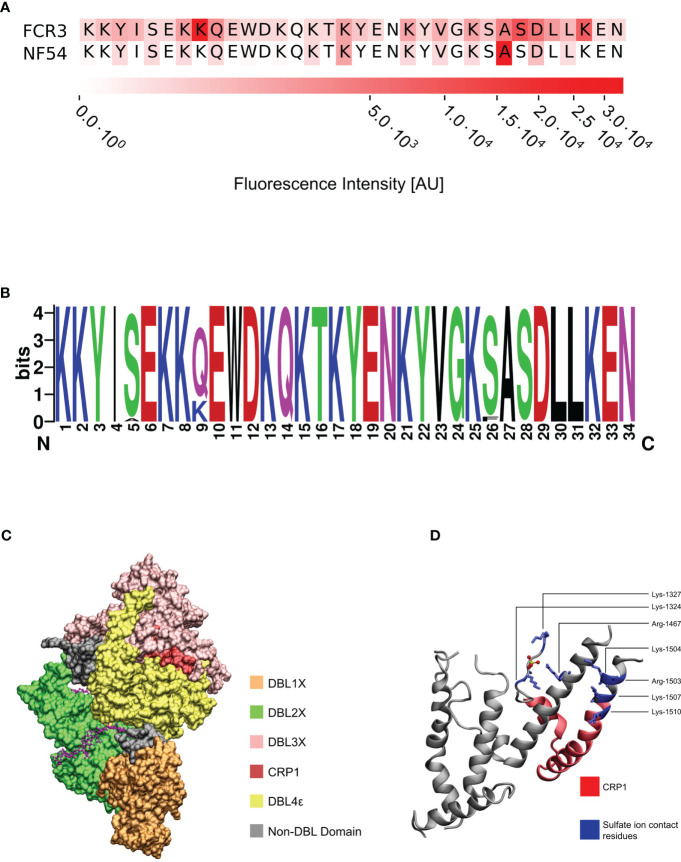
3D10 recognizes a conserved epitope in VAR2CSA. **(A)** Sequence alignment of the most reactive epitope in the peptide arrays. **(B)** Logo plot of 722 VAR2CSA sequences across Africa and Asia depicting the degree of conservation at each amino acid position of the epitope. Color scheme is based on their chemical properties: polar = green, basic = blue, acidic = red, hydrophobic = black. **(C)** Cryo-EM of the core structure of NF54 VAR2CSA (DBL1-DBL4; PDB: 7JGH) in the presence of chondroitin sulfate A (magenta). **(D)** Crystal structure of the VAR2CSA DBL3X domain depicting the specific residues (blue) that interact with the sulfate ion from CSA (PDB: 3CPZ). The epitope from **(A)** is colored red.

We screened peptide arrays spanning the ectodomain of VAR2CSA from the FCR3 and NF54 alleles to identify epitopes recognized by 3D10 (**Supplementary Figure 1**). Based on the recognition of multiple overlapping peptides and the intensity of the antibody binding signal, 3D10 recognized three stronger epitopes and several weaker epitopes throughout the protein. The most reactive epitope in both arrays maps to a region in DBL3X (**Figure 1**). The amino acids in this epitope are 100% conserved between the two alleles (**Figure 1A**) and extremely well-conserved in VAR2CSA sequences from over 700 field isolates, with polymorphisms at only four residues (positions 1513, 1517, 1534, and 1536) (**Figure 1B**). Based on the cryo-EM structure of VAR2CSA, this epitope maps to a region that is distinct from the major CSA binding channel (**Figure 1C**). However, the epitope does map to a previously reported CSA binding region within subdomain 3 of DBL3X (Singh et al., 2008; Singh et al., 2010). Specifically, it is adjacent to six of the amino acid residues that are predicted to interact with CSA (**Figure 1D**; blue) and includes two residues, Lys-1510 and Lys-1515, that are important for DBL3X binding to CSA (Singh et al., 2008; Khunrae et al., 2009).

**Figure 2:**
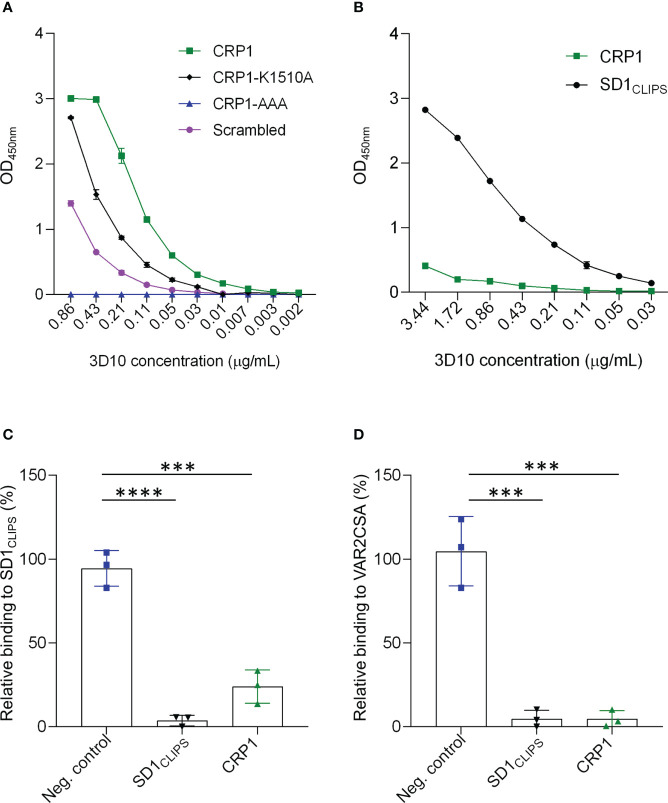
3D10 targets an epitope shared between SD1_CLIPS_ and CRP1. **(A)** Titration of 3D10 against CRP1 and its variants coated at 5 µg/ml. **(B)** Titration of 3D10 against CRP1 and SD1_CLIPS_ coated at 1 µg/ml. In **(A, B)**, OD values were subtracted from the OD of the isotype control immunoglobin G1 (IgG1) and presented as relative OD. Data are mean ± SD. **(C, D)** Competition ELISA of 3D10 with SD1_CLIPS_ **(C)** or VAR2CSA **(D)** as the capture antigen. Competing antigens are an unrelated peptide (neg. control; blue), SD1_CLIPS_ (black) and CRP1 (green). Data are the mean ODs with competitor relative to no competitor ± SD of 3 independent experiments. Significance was determined using a one-way ANOVA with Tukey’s multiple comparisons test (***P ≤ 0.0002; ****P < 0.0001).

To further characterize our lead epitope, we synthesized a peptide, called CRP1, that includes 34 residues from the target region in VAR2CSA. We confirmed that 3D10 recognizes this peptide by ELISA (**Figure 2A**). A scrambled version of CRP1 was poorly recognized by 3D10. To further define the epitope, we tested 3D10 recognition of CRP1 mutants where the following lysine residues were mutated to alanine: CRP1-K1510A, CRP1-AAA (mutations at positions 1521, 1525 and 1529). For the triple mutant, we hypothesized that 3D10 may recognize a specific topology based on the pattern of lysines along the face of the alpha helix in CRP1 (**Figure 1D**). Indeed, the three mutations completely abolished antibody recognition while mutation of the K1510 partially reduced binding to 3D10.

Next, we compared the level of recognition of 3D10 to CRP1 and SD1_CLIPS_, which is a structurally constrained peptide from subdomain 1 (SD1) of PvDBP, the natural epitope for 3D10. When both peptides were coated at the same concentration (1 μg/ml; 5 times lower than in **Figure 2A**), the cross-reactivity of 3D10 to the heterologous CRP1 peptide was substantially weaker compared to the homologous SD1_CLIPS_ peptide, as expected (**Figure 2B**). Nevertheless, the recognition of CRP1 by 3D10 suggests an epitope is shared between CRP1 and SD1_CLIPS_. We tested this by competition ELISA using CRP1 as the competitor and SD1_CLIPS_ as the capture antigen (**Figure 2C**). The 3D10 MAb was preincubated either with CRP1, SD1_CLIPS_ as a positive control, or an unrelated peptide (P10) as a negative control, before adding the mixtures to wells coated with SD1_CLIPS_. We observed a significant loss in SD1_CLIPS_ recognition when competing with CRP1 compared with the negative control peptide (*P* = 0.0001; **Figure 2C**). These results indicate that although the binding of 3D10 to CRP1 is weaker than for SD1_CLIPS_, the peptide from VAR2CSA can compete with the PvDBP peptide for binding to 3D10.

We used a similar competition ELISA strategy to confirm that 3D10 recognizes VAR2CSA *via* CRP1 (**Figure 2D**). As we reported previously, SD1 peptide competed with VAR2CSA for binding to 3D10 (Mitran et al., 2019) and this served as our positive control. When we competed with CRP1, we observed the same binding inhibition toward VAR2CSA as compared to SD1_CLIPS_. Thus, CRP1 is a major epitope in VAR2CSA targeted by 3D10.

### CRP1 binds to soluble CSA and elicits adhesion-blocking antibodies

**Figure 3:**
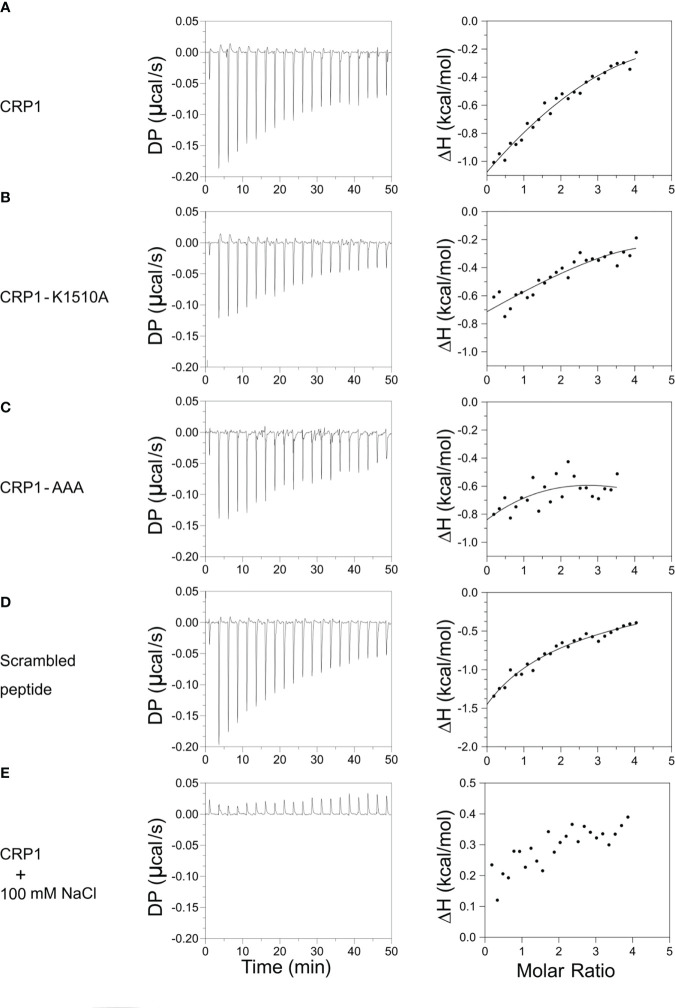
CRP1 and its mutants bind to CSA. The left panels depict heat exchange during the binding events between the peptides and CSA. The right panels show the change in enthalpy plotted against the molar ratio of CSA to peptides. Binding isotherms of **(A)** CRP1, **(B)** CRP1-K1510A, **(C)** CRP1-AAA, **(D)** scrambled peptide, and **(E)** CRP1 in the presence of 100 mM NaCl. DP – Differential power (μcal/s), ΔH – enthalpy change (kcal/mol).

In addition to recognizing recombinant VAR2CSA, the 3D10 MAb blocks binding of VAR2CSA-expressing IEs to CSA in the IBA (Gnidehou et al., 2019). The fact that CRP1 maps to the site within DBL3X purported to bind CSA and includes the key lysine residues at position 1510 and 1515 suggests that 3D10 may directly interfere with CSA binding or cause steric hindrance. To address this, we measured the binding interaction of CRP1 and CSA using ITC. We observed heat release after each CSA injection, consistent with CSA binding to CRP1, and a typical sigmoidal exothermic binding isotherm due to this interaction (**Figure 3A**). To determine if the lysines that are important for 3D10 recognition also contribute to binding to CSA, we measured the binding interaction of the CRP1 mutants, CRP1-K1510A and CRP1-AAA. We observed a similar binding pattern as the wild type CRP1 (**Figures 3B, C**, respectively). Further, the scrambled CRP1 peptide also bound to CSA (**Figure 3D**). Binding was abrogated when CRP1 was incubated with CSA in the presence of 100 mM NaCl (**Figure 3E**). These data suggest that CRP1 interacts with CSA via electrostatic interactions.

**Figure 4:**
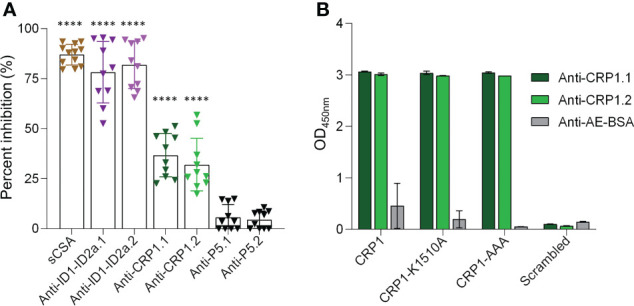
Anti-CRP1 IgG inhibits IE binding to CSA. **(A)** The percent inhibition was calculated from the number of IEs bound to CSA after pre-incubation with 200 µg/ml of anti-CRP1 IgG compared with 200 µg/ml IgG from rats immunized with the carrier protein conjugate. Controls for the binding assay included soluble CSA (sCSA) and IgG raised against ID1-ID2a (PAMVAC vaccine construct) as positive controls, and an unrelated peptide (P5) as a negative control. Two rats were immunized for each immunogen and denoted as ‘.1’ and ‘.2’. Data are mean ± SD from three independent experiments and analyzed with one-way ANOVA followed by Tukey’’s multiple comparisons test (****P < 0.0001 for each dataset relative to the anti-P5 controls). **(B)** Reactivity of rat sera (1/100 dilution) against CRP1, CRP1 mutants and a scrambled peptide generated based on the CRP1 sequence. Sera from rats immunized with only the carrier protein was used as a negative control. Anti-AE-BSA represents pooled sera from two rats.

Given the association of CRP1 with CSA and the blocking activity of the 3D10 MAb, we asked if anti-CRP1 antibodies can inhibit the binding of IEs to CSA. Antibodies to the CRP1-BSA conjugate were raised in outbred rats, and total IgG from each animal (‘anti-CRP1.1’ and ‘anti-CRP1.2’) was tested in the IBA against the *P. falciparum* CS2 strain that was selected for high expression of VAR2CSA and binding to CSA (**Figure 4A**; **Supplementary Figure 2**). Soluble CSA and IgG raised against the construct used in the PAMVAC vaccine (spanning ID1-ID2a in VAR2CSA) were used as positive controls and blocked over 90% of IEs from binding to CSA. As our comparator, rats were immunized with another unrelated peptide conjugated to BSA (P5-BSA) that did not display inhibitory activity in the IBA. We observed significant inhibition (32-36%) with IgG from both CRP1-BSA immunized rats compared to P5-BSA IgG, indicating that CRP1 can elicit antibodies with similar blocking activity to 3D10. To evaluate the specificity of the rat-CRP1 antibodies, we tested these antibodies against CRP1, CRP1 mutants, and the scrambled CRP1 sequence. The rat-CRP1 antibodies recognized CRP1 and the mutants but did not recognize the scrambled peptide (**Figure 4B**).

### Seroreactivities to CRP1 and SD1_CLIPS_ are strongly correlated in Colombian cohorts

**Figure 5:**
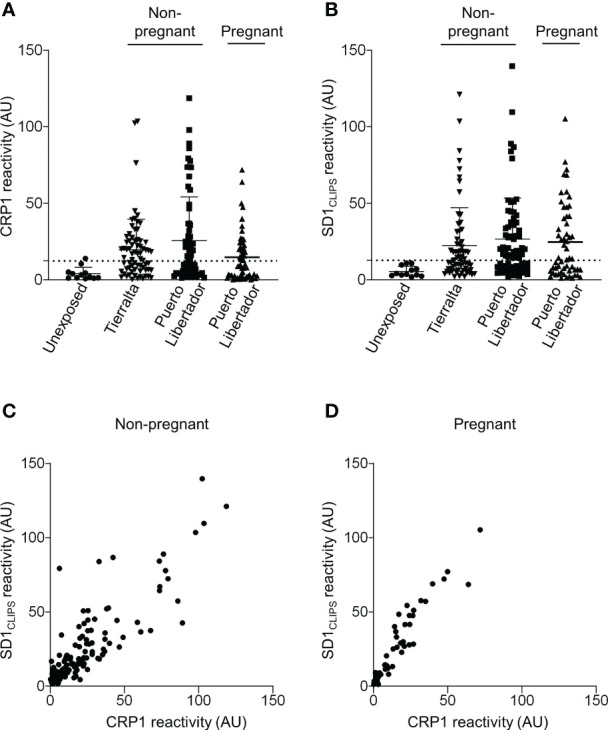
Seroreactivity to CRP1 correlates strongly with SD1 reactivity. **(A)** Sera from different populations in Colombia were tested by ELISA for reactivity to CRP1 (1/400 dilution), and **(B)** SD1_CLIPS_ (1:200 dilution). Sera from malaria-exposed populations were selected based on VAR2CSA seropositivity. Antibody binding (OD) was converted to arbitrary units (AU) based on the positive control included on every plate. Values are expressed as mean AU ± SD. The dashed line represents the cut-off for CRP1 and SD1_CLIPS_ seropositivity based on the mean AU (+2 SDs) from sera of individuals living in a non-endemic region of Colombia. **(C)** Serum reactivity against CRP1 and SD1_CLIPS_ was correlated using Spearman rank correlation in the non-pregnant cohorts from Puerto Libertador and Tierralta (r_s_= 0.8424, *P*<0.0001, n=137), and **(D)** pregnant populations (r_s_= 0.9449, *P*<0.0001, n=56). r_s_, Spearman rank coefficient.

In our previous studies in Colombia, we reported that SD1-specific human antibodies could compete with 3D10 for recognition of VAR2CSA by ELISA (Mitran et al., 2019). As CRP1 is a major epitope recognized by 3D10, this epitope may also be the target of SD1-specific human antibodies. We therefore tested whether VAR2CSA-seropositive samples from our Colombian cohorts recognized CRP1. We reported previously the frequency of VAR2CSA seropositivity in pregnant and non-pregnant populations residing in Puerto Libertador (Gavina et al., 2018; Gnidehou et al., 2019). In our recent cross-sectional study of men and children in Tierralta, 75% of participants had antibodies to VAR2CSA (70/93). We observed high reactivity to CRP1 in these two cohorts: 50.7% in Puerto Libertador (34/67) and 57% in Tierralta (40/70) (**Figure 5A**). Antibodies to CRP1 were also frequently detected (46%) in the VAR2CSA-seropositive pregnant women from Puerto Libertador (26/56). In contrast, among the negative control samples from individuals living in Medellín with no exposure to malaria, only one of the 12 samples (8.3%) had low reactivity to CRP1.

We previously observed a correlation between reactivity to SD1 and VAR2CSA in Colombian non-pregnant populations (Mitran et al., 2019). If CRP1 is a major target of cross-reactive antibodies that arise from exposure to SD1 (from PvDBP), we would expect reactivity to the two epitopes in PvDBP and VAR2CSA to correlate. Overall, the frequency of antibodies to SD1_CLIPS_ was similar to CRP1 in all cohorts (50.7% (34/67) and 62.8% (44/70)) in the non-pregnant populations, and 53.6% in the pregnant women (30/56); **Figure 5B**). Further, antibody levels to CRP1 and SD1_CLIPS_ were strongly correlated in sera from the non-pregnant population (r_s_ =0.8424, *P*<0.0001) (**Figure 5C**) and pregnant women (r_s_ =0.9449, *P*<0.0001) (**Figure 5D**).

## Discussion

Despite the evidence that VAR2CSA antibodies are acquired only during pregnancy, we and others reported VAR2CSA antibodies in men and children (Gnidehou and Yanow, 2021). We proposed that these antibodies can arise from exposure to PvDBP, which contains a structurally conserved DBL domain (Howell et al., 2006). In this study, we used the 3D10 MAb to determine the epitopes in VAR2CSA that are recognized by a PvDBP antibody. The strongest epitope, CRP1, maps to DBL3X. Two other epitopes were strongly recognized, including one that maps to a region of DBL5ε that we identified in a previous peptide array of this domain (Mitran et al., 2019). This epitope was not pursued further because it is polymorphic. We also noted several weaker epitopes, but their relevance is not yet clear. From these array data, we infer that the MAb 3D10 is somewhat promiscuous in its reactivity, recognizing peptides with very limited sequence homology but that share other features. This is apparent when comparing the sequences of CRP1 with SD1, the natural target of 3D10 in PvDBP. The sequence similarity is only 24% and yet the two peptides compete for recognition of 3D10. Rather, the shared epitope may consist of positively charged residues, particularly lysines and arginines. In SD1, mutation of the amino acids ‘NxxRKR’ abolished recognition by 3D10 (Chen et al., 2016), while in CRP1, we showed that three of the lysines are critical for 3D10 recognition and a fourth lysine (1510) is important.

The CRP1 epitope recognized by 3D10 maps to the positively charged patch in subdomain 3 (SD3) of DBL3X that interacts with CSA (Higgins, 2008; Singh et al., 2008; Khunrae et al., 2009; Singh et al., 2010). Here, we showed that the CRP1 peptide can interact directly with CSA. Based on published results that K1510 is important for the interaction of DBL3X with chondroitin sulfate proteoglycan (Singh et al., 2008; Khunrae et al., 2009), we expected the K1510A mutant would not bind to CSA, but it did. One explanation is that this amino acid may contribute to CSA binding within the context of a larger protein structure (DBL3X), but the interactions of the free peptide with CSA, albeit primarily electrostatic, could involve other residues. This is consistent with our observation that the scrambled peptide and the CRP1-AAA mutant peptide also bound to CSA. Despite lack of specificity in the interactions between the CRP1 peptide and CSA by ITC, rat antibodies to CRP1 blocked CSA binding of IEs that express native VAR2CSA. Since CRP1 includes two amino acids implicated previously in binding of DBL3X to CSA and is adjacent to the other CSA-interacting residues, we propose that anti-CRP1 IgG block binding through steric hindrance of a binding site in DBL3X. Although cryo-EM structures of VAR2CSA complexed with CSA clearly identified a major binding channel formed by the NTS, DBL1X, DBL2X and DBL4ε domains (Ma et al., 2021; Wang K. et al., 2021; Wang W. et al., 2021), prior studies demonstrated that the DBL3X domain contains epitopes targeted by inhibitory antibodies (Nielsen et al., 2009; Srivastava et al., 2011; Obiakor et al., 2013). Consistent with these findings, rat sera raised against the entire DBL3X domain blocked binding of IEs to CSA and these antibodies strongly recognized peptides spanning CRP1 (Salanti et al., 2010).

The CRP1 sequence may present certain properties for glycan binding that are shared among other parasite proteins. For example, the minimal CSA binding region identified in the DBLγ of var1CSA (FCR3varCSA) is closely related to CRP1 and rabbit antisera to this sequence inhibits parasite binding to CSA (Gamain et al., 2004). An intriguing observation is that the SD3 of DBL3X has a similar structure to the corresponding regions in the DBL domains of EBA-175 (F1 and F2) and Pkα-DBL (Dahlbäck et al., 2006; Higgins, 2008). Alignment of these DBL domains shows that Ile-1512 and Lys-1516 from CRP1 are shared and two other amino acids (Tyr-1511 and Trp-1519) have conserved substitutions (Andersen et al., 2008; Singh et al., 2008). Given the structural conservation of DBL domains in *Plasmodium*, antibody cross-reactivity to these epitopes could extend beyond our observations with PvDBP and VAR2CSA.

The biological relevance of cross-reactive antibodies is not yet clear but in the case of VAR2CSA, characterization of these antibodies can help identify new epitopes for vaccine design. In our studies from Colombia, we reported antibodies in non-pregnant populations that recognized VAR2CSA and had modest inhibitory activity in the IBA (Gnidehou et al., 2019). In trying to understand the origin of these antibodies, we discovered that these antibodies correlated with reactivity to SD1 from PvDBP and that SD1 affinity-purified antibodies blocked parasite binding to CSA by around 32-45% (Mitran et al., 2019). Here we explored whether CRP1 is the target of these cross-reactive antibodies and observed a high frequency of CRP1 antibodies that strongly correlated with reactivity to SD1. Given that SD1 affinity-purified antibodies and 3D10 competed for recognition of VAR2CSA (Mitran et al., 2019), we propose that exposure to SD1 can elicit antibodies that recognize CRP1 and block parasite binding to CSA *via* the putative binding site in DBL3X. In Colombian pregnant women, these antibodies to CRP1 may contribute to protection against *P. falciparum* in pregnancy, but this must be evaluated in prospective clinical studies. While cross-reactive antibodies from PvDBP may be one source of antibodies to CRP1, they could also arise from natural exposure to VAR2CSA in pregnancy. Plasma from pregnant Ghanian women had antibodies that recognized epitopes within the CRP1 region in DBL3X (Dahlbäck et al., 2006). Given the extremely high conservation of CRP1 in field isolates (**Figure 2B** and (Dahlbäck et al., 2006)), it is not likely to be an immunodominant epitope in VAR2CSA but it may elicit antibodies during infection that could be boosted by subsequent infection with other strains of *P. falciparum* in pregnancy. This feature, and the inhibitory activity of CRP1 antibodies *in vitro*, suggest this epitope may be a valuable complement to existing VAR2CSA vaccines that target the major CSA binding channel (Ma et al., 2021; Wang K. et al., 2021; Wang W. et al., 2021). Such a vaccine could target multiple CSA-binding sites in VAR2CSA to mitigate parasite immune evasion strategies and elicit strain-transcending immunity against placental malaria.

## Data availability statement

The datasets presented in this study can be found in online repositories. The names of the repository/repositories and accession number(s) can be found below: https://github.com/Yanow-lab/Iyamu-et-al.

## Ethics statement

The studies involving human participants were reviewed and approved by Health Research Ethics Board of the University of Alberta, Canada (approval Pro00041720), the Comité de Ética of Instituto de Investigaciones Médicas of Universidad de Antioquia, Colombia (approvals 009-2013, 002-2015, 009-2016 and 003-2020), Institutional Review Boards at the School of Public Health, Makerere University College of Health Sciences (The Higher Degrees, Research and Ethics Committee – HDREC), and the Uganda National Council of Science and Technology (UNCST) (HDREC 368). Written informed consent to participate in this study was provided by the participants’ legal guardian/next of kin. Ethical review and approval was not required for the animal study because Animal immunizations were performed commercially.

## Author contributions

UI, SY, and BH conceived the study, and the manuscript was written by UI and SY. Experiments and data analyses were performed by UI, DV, BT, SG, KM, RB, and TA. Human sera from Colombia were provided by EA and AM. MO supervised the ITC experiments. All authors contributed to the article and approved the submitted version.
