# Supplementary material for: A conserved epitope in VAR2CSA is targeted by a cross-reactive antibody originating from Plasmodium vivax Duffy binding protein
Source: Front Cell Infect Microbiol. 2023 Jun 16;13:1202276. doi: 10.3389/fcimb.2023.1202276 (PMC10312377; doi:10.3389/fcimb.2023.1202276)
Supplement: Supplementary file 1 [file DataSheet_1.pdf]

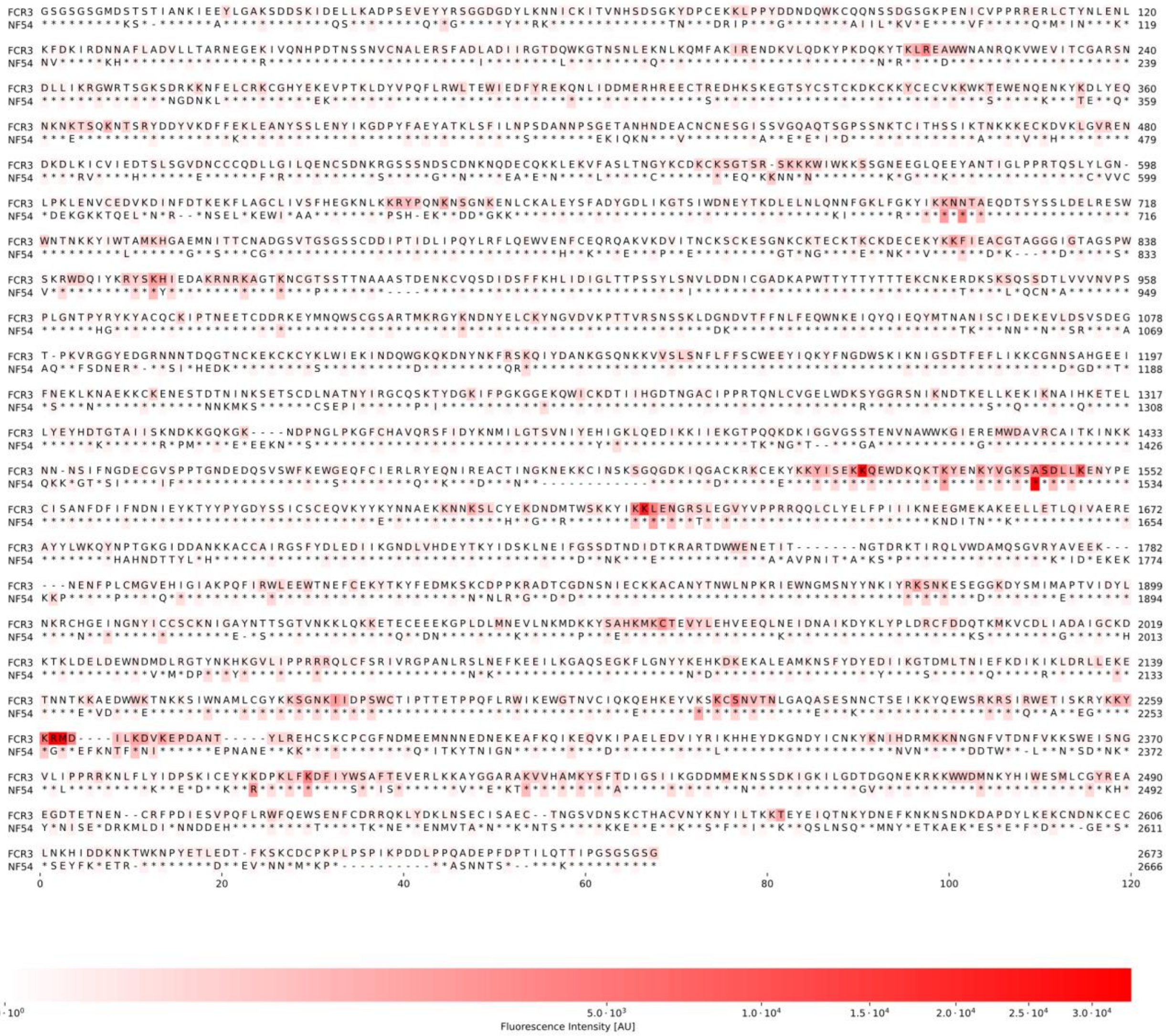

FIGURE S1. Mapping of epitopes in VAR2CSA recognized by 3D10. Sequence alignment of the FCR3 and NF54 ectodomains of VAR2CSA. Stars indicate amino acids that are identical between the two alleles. A dash corresponds to a gap in the sequence alignment. Arrays of 20-mer peptides with 19 or 18 amino acid overlap (FCR3 and NF54, respectively) were screened with 3D10. Fluorescence intensity of antibody binding to each peptide was measured in arbitrary units (AU) and coloured according to the red scalebar. Colours were assigned to the last amino acid in the peptide.

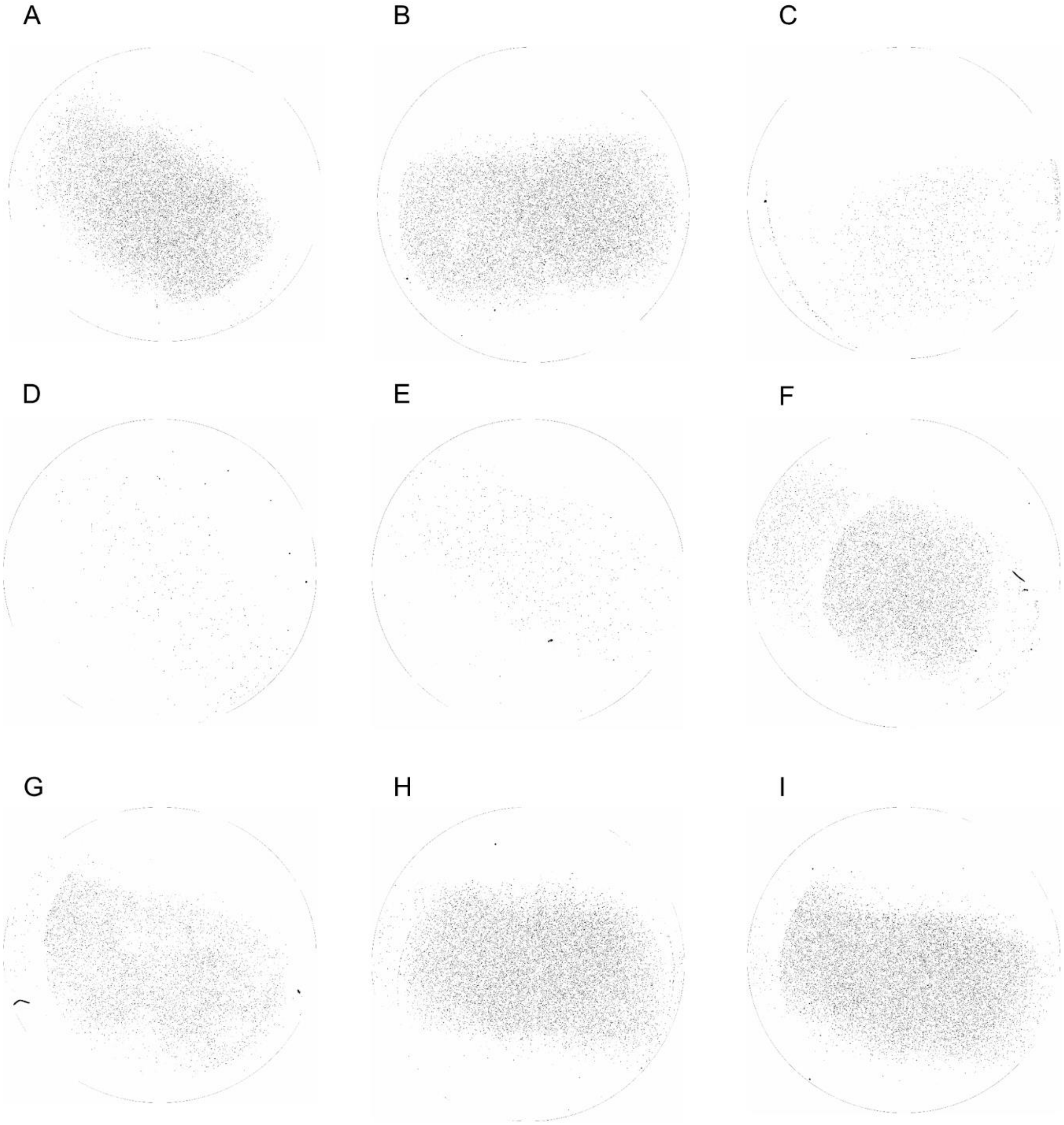

FIGURE S2: Anti-CRP1 IgG inhibits IE binding to CSA. IEs bound to plates spotted with CSA after pre-incubation with AE-BSA (A), PBS (B), sCSA (C), anti-ID1-1D2a.1 and anti-ID1-1D2a.2 IgG (D, E), anti-CRP1.1 and anti-CRP1.2 IgG (F,G), and anti-P5.1 and anti-P5.2 IgG (H,I). Each spot is a representative image from an experiment performed on the same day. sCSA – Soluble chondroitin sulfate A (CSA).
